# On unsteady 3D bio-convection flow of viscoelastic nanofluid with radiative heat transfer inside a solar collector plate

**DOI:** 10.1038/s41598-022-06728-0

**Published:** 2022-02-22

**Authors:** Umar Farooq, Hassan Waqas, Zahir Shah, Poom Kumam, Wejdan Deebani

**Affiliations:** 1 Department of Mathematics, Government College University Faisalabad, Faisalabad, 38000 Pakistan; 2 Department of Mathematical Sciences, University of Lakki Marwat, Lakki Marwat, 28420 Khyber Pakhtunkhwa Pakistan; 3 Fixed Point Research Laboratory, Fixed Point Theory and Applications Research Group, Center of Excellence in Theoretical and Computational Science (TaCS-CoE), Faculty of Science, King Mongkut’s University of Technology Thonburi (KMUTT), 126 Pracha Uthit Rd., Bang Mod, Thung Khru, Bangkok, 10140 Thailand; 4 Department of Medical Research, China Medical University Hospital, China Medical University, Taichung, 40402 Taiwan; 5 Department of Mathematics, College of Science and Arts, King Abdulaziz University, P.O. Box 344, Rabigh, 21911 Saudi Arabia

**Keywords:** Engineering, Mathematics and computing, Nanoscience and technology, Physics

## Abstract

Nanoparticles are used in industrial and engineering by allowing for faster heat transfer rates in microchips, vehicle cooling, food processing, and nuclear reactors. This research aims to scrutinize the three-dimensional bioconvectional flow performances of viscoelastic nanofluids through a elongating sheet with motile microorganisms. Radiative impact and solutal boundary conditions are studied here. The impacts of thermophoresis, Brownian motion, and bioconvection are also considered. By using suitable similarity transformations, the PDEs are converted into ODEs. The numerical and graphical results are calculated with the help of shooting scheme built-in function Bvp4c in computational tool MATLAB. The graphical and numerical importance of physical engineering parameters like local skin friction, local Nusselt, local Sherwood, and local motile microorganism numbers are discussed here. The thermal profile is enhanced for the higher estimations of the Brownian motion and thermophoresis parameter. The heat profile is boosted up for the increasing variations of the thermal radiation and the thermophoresis parameter. The energy profile is improved by increasing the estimations of solutal Biot number while declining for mixed convection parameter and unsteadiness parameter. The microorganism profile decays for Peclet and bioconvection Lewis number while rising for buoyancy ratio parameter and bioconvection Rayleigh number.

## Introduction

**Figure 1:**
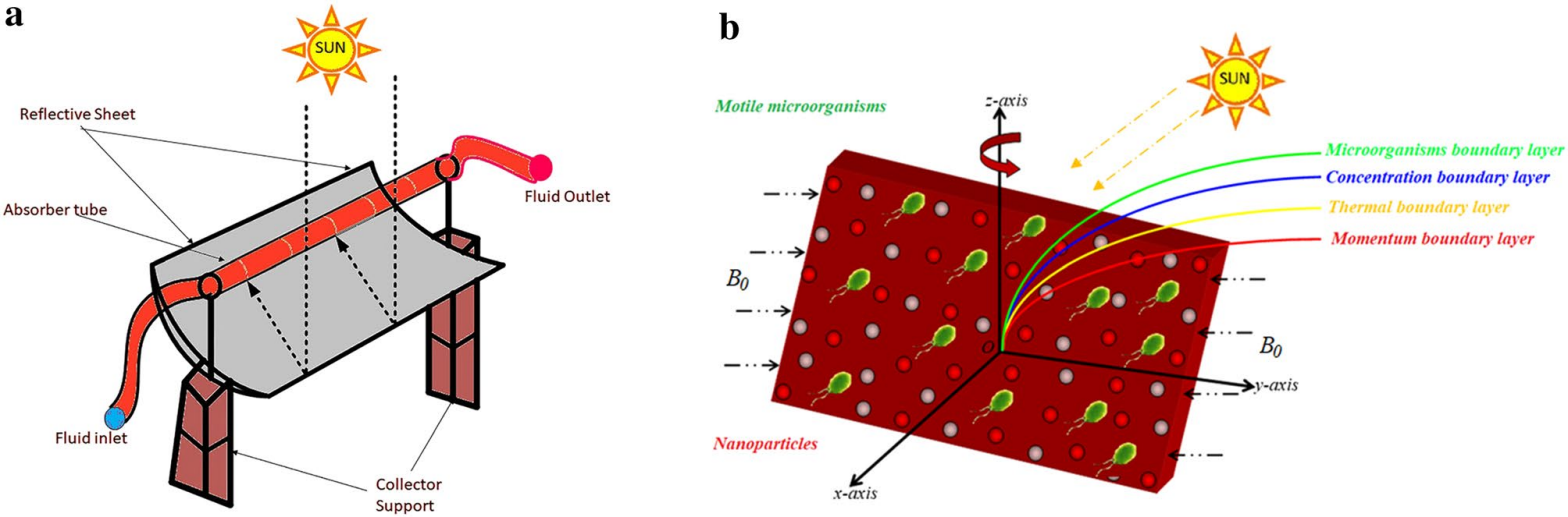
(**a**) Solar effects. (**b**) Physical sketch of the problem.

Because of their many uses in engineering and science, nanofluids pique the interest of many researchers. Nanostructures are well-known as heat transfer coolants and are often used to avoid mobile system overheating. Furthermore, nanofluid cleaning products, micro-cryosurgery, automotive evolve from biological and environmental, and computer chips use nanofluids. Choi^1^ proposed the central principle of nanoliquid in 1995, which has since been expanded upon by many researchers. Buongiorno^2^ studied nanomaterials convection transfer by taking Brownian and thermophoresis diffusion properties into account. Hsiao^3^ numerically investigated micropolar nanofluid flow with multifaceted properties such as viscous diffusion and magnetic field. Turkyilmazoglu^4^ investigated the significance of nanomaterials in an asymmetrical channel using the well-known Buongiorno model. Alblawi et al.^5^ used the Buongiorno model of curve extended geometry to investigate nanoparticles in thermo physics. Khan and Pop^6^ investigated the movement of nanofluids through the Buongiorno model after it had gone via the stretched surface. Multiwall carbon nanotubes in nanofluid boundary layer flow were investigated by Shafiq et al.^7^. Hayat et al.^8^ investigated the diffusion implications on the transportation of viscoelastic nanofluids via extensible walls. The flow of nanoliquid in porous media was studied by Hayat et al.^9^. By changing the structure, Azam et al.^10^ studied the characteristics of Carreau nanoliquid flow. Elgazery^11^ deals with the theoretical connection of four different types of nanoparticles characteristics. Ahmad et al.^12^ investigated three-dimensional nanofluid flow with Brownian motion and thermophoresis. The flow of the Buongiorno model through a conductive and considerably expanded network was reported by Alblawi et al.^5^. Hayat et al.^13^ used nanomaterials power to study the flow of MHD nanofluids across a non-linear surface. Asma et al.^14^ looked at 3D nanofluid magnetized flow caused by energy activation and heat generation/absorption caused by a spinning disc issue. Eid et al.^15^ studied the Carreau nanofluid flow above a nonlinear moving surface using chemical and heat absorption/generation techniques. Al-Bashir et al.^16^ investigated cell heat and solar radiation. Sohail et al.^17^ studied the fluid in the presence of thermal radiations. Nader et al.^18^ investigated a photovoltaic system with a refrigeration system. Hussien et al.^19^ conducted a brief study into the heat transfer of nanofluids. Abu-Libdeh et al.^20^ investigated the entropy of a hybrid nanofluid on a highly permeable cavity. Hussien et al.^21^ explored the heat transfer of hybrid nanofluids in the presence of thermal radiation. Al-Kouz et al.^22^ investigated the role of heat transfer with nanocomposites in chemical reactions. Mahesh et al.^23^ looked into the importance of hybrid Nanofluid flow over discs with entropy generation enhancement. Mahanthesh et al.^24^ investigated the aspects of quadratic convection on dusty nanofluid on a vertical surface. Owhai et al.^25^ investigated the effects of a given prescription heat flux on nanoliquid. Attempts^26–33^ indicate investigators' additional research in the field of nanofluids. Over the last decade, through use of nanofluids in solar collectors has gotten a lot of scientific interest. Nevertheless, there are significant gaps in the research on the use of oil-based nanoliquid in solar panels, as there isn't enough material, particularly practical studies, available. This work aims to add to the existing body of knowledge by demonstrating the results of the two new oil-based hybrid nanofluids in a solar collector. The thermal efficiency and heat transmission capabilities of the nanofluids were assessed. The research provides a green approach to the synthesis of nanomaterials for application in solar collectors. OLE-ZVI and OLE-TiO_2_ are two nanoparticles made from the almond extract of leaves. The impacts of nanofluids on exert economic efficiency, thermal expansion, and hydraulic gradient anywhere along PTC’s suction pipe are included in a performance assessment of the collector. In addition, we offer a comparison of our findings to those available in the literature. A reflector sheet curved in a parabola form and a receiver’s tube positioned at a focal length from either the reflected sheet make up the collectors. The reflecting sheet (mirror) collects direct solar energy and concentrates it on the receivers, which are positioned at the parabola's center. This procedure is depicted in diagram form in Fig. 1a. A heat exchanger flows through the receiver tube, transferring the photovoltaic solar energy to the fluid via temperature distribution. The fluid transports the usable energy to the diverse applications in which it is employed.

Bioconvection is known as a microorganism swimming in a particular direction that is heavier than water, resulting in a gradient of density that can contribute to the formation of a macroscopic convective motion. Bioconvection is a technique used in nanomaterials and bioengineering that has the potential to improve mass transport and induce mixing, especially in micro volumes that can be used to create a stable flow of nanofluids. As a result, primary-level bioconvection research in nanofluids may aid in the development of effective microfluid systems. Furthermore, bioconvection is crucial in mechanical engineering because the electrical field is understood to regulate the bioconvection phenomenon for the output of energy or mechanical power. Avramenko^34^ observed bioconvection when suspending miniature life forms. Bees et al.^35^ investigated “bioconvection”, which depicts hydrodynamic unsteadiness and examples of living body liquids. Khan et al.^36^ examined the logical formation of silver nanoparticles over a given base liquid and the resulting nanofluids that create the base liquids’ warmth limit. Tlili et al.^37^ investigated the slip and activation-energy activities of Oldroyd-B nanofluids during bioconvection. Hassan et al.^38^ investigated statistical mechanisms for transferring nanomaterials in liquid on a rotating disc with slip impacts. Shafiq et al.^39^ investigated the bioconvection properties of a nanofluid under convective flow. Khan et al.^40^ investigated the bioconvection consequences of nanoliquid. The capacity of charged digression magnetic nanofluids to engage with motile microorganisms was explored by Wang et al.^41^. The movement of tangent hyperbolic boundary layer flow microorganisms with bioconvection processes was studied by Shafiq et al.^42^. Khan et al.^43^ used an oscillatory stretchy layer to examine the relevance of nanofluid bioconvection motion. The study of motile microorganisms, such as their movement variations, is based on their reactions to various science, industrial, fiscal, and social elements. More work on bioconvection is carried out^44–56^.

The main purpose of the research is to investigate the effects of bioconvection and thermal radiation in the 3D flow of a viscoelastic nanofluid containing motile microorganisms above a stretching surface. The incorporation of motile microorganisms into nanomaterials is critical for increasing the thermal performance of a wide range of structures, including bacterial energy units, chemical biosensors, and bio-micro processes. The problem was discussed in terms of partial differential equations with suitable boundary conditions, which were then converted into a set of partial differential equations with non-similar variables. This provides control of PDEs was numerically solved using local non-similarity and the bvp4c shooting method. Graphs have been used to evaluate the aspect of the critical parameters on the flow field. Physical quantities including skin friction, local Sherwood, the local Nusselt, and the local motile microorganism number are tabulated with different physical parameter values. The ranges of the physical flow parameters like as $0.5 \le \Pr \le 2.0$, $0.1 \le Rd \le 1.5$, $0.0 \le \alpha \le 1.2$, $0.2 \le \gamma_{1} \le 0.8$, $0.5 \le Nb \le 2.0$, $0.5 \le Nt \le 2.0$, $0.1 \le Nr \le 1.5$, $0.1 \le Nc \le 1.2$, $0.1 \le \lambda \le 1.2$, $0.0 \le S \le 0.6$, $1.0 \le Le \le 2.0$, $0.2 \le \gamma_{2} \le 0.8$, $0.1 \le \beta \le 1.5$, $0.2 \le \gamma_{3} \le 0.8$, $0.1 \le {\text{Pe}} \le 1.0$ and $1.0 \le Lb \le 2.0$ are discussed.

### Mathematical formulation

Here we investigated the significance of Brownian motion, thermophoresis effects, and thermal radiation over a three-dimensional viscoelastic fluid containing swimming motile microorganisms over a stretched surface. For nanofluid, the Buongiorno model is being investigated. The bioconvection phenomenon is taken into account. We use a Cartesian coordinate system with extended *x* and *y*-axis coordinates and a *z*-axis perpendicular to the surface (see Fig. 1b). The stretching surface velocities along $x$ the axis and $y$ axis are introduced as $U_{w} \left( x \right) = \frac{ax}{{1 - ct}}$ & $V_{w} \left( y \right) = \frac{by}{{1 - ct}}$ respectively.

The flow equations are defined as

1 $$\left. {\begin{array}{*{20}l} {\nabla \cdot V = 0,} \hfill \\ {\rho_{f} V_{t} = - \rho_{f} V \cdot \nabla V - \nabla p + \nabla .\sigma } \hfill \\ {\left( {\rho c_{p} } \right)\left( {T_{t} + V \cdot \nabla T} \right) = \alpha_{m} \nabla^{2} T + \tau \left( {D_{B} \nabla T \cdot \nabla C + \frac{{D_{T} }}{{T_{\infty } }}\nabla T \cdot \nabla T} \right) - 1/\left( {\rho c} \right)_{f} D_{z} q_{r} } \hfill \\ {\left( {\rho c_{p} } \right)\left( {C_{t} + V \cdot \nabla C} \right) = D_{B} \nabla^{2} C + \left( {\frac{{D_{T} }}{{T_{\infty } }}} \right)\nabla^{2} T,} \hfill \\ \end{array} } \right\},$$

2 $$N_{t} + V \cdot {\text{J}}_{0} = 0,$$

where

3 $$\begin{aligned} & J_{0} = NV + N\hat{V} - D_{m} \nabla N, \\ & where \\ & \hat{V} = \left( {{\raise0.7ex\hbox{${bW_{c} }$} \!\mathord{\left/ {\vphantom {{bW_{c} } {\Delta C}}}\right.\kern-\nulldelimiterspace} \!\lower0.7ex\hbox{${\Delta C}$}}} \right)\nabla C. \\ \end{aligned}$$

In which $\left( V \right)$ is velocity vector, $\left( {\alpha_{m} } \right)$ is thermal diffusivity, $\left( {J_{0} } \right)$ is the convection of liquid, and $\left( {\hat{V}} \right)$ cell swimming speed. Under considered assumptions, governing expressions related to conservations of mass, momentum, energy, volumetric concentration, and motile micro-organisms are described into following relations^12,39^:

4 $$u_{x} + v_{y} + w_{z} = 0,$$

5 $$\begin{aligned} & u_{t} + uu_{x} + vu_{y} + wu_{y} = \nu u_{zz} + k_{0} \left( {u_{zzt} + uu_{zzx} + wu_{zzz} - u_{x} u_{zz} - u_{z} w_{zz} - 2u_{z} u_{xz} - 2w_{z} u_{zz} } \right) \\ & \quad + \;\frac{1}{{\rho_{f} }}\left[ {\left( {1 - C_{f} } \right)\rho_{f} \beta^{**} \left( {T - T_{\infty } } \right) - \left( {\rho_{p} - \rho_{f} } \right)g^{*} \left( {C - C_{\infty } } \right) - \left( {N - N_{\infty } } \right)g^{*} \gamma \left( {\rho_{m} - \rho_{f} } \right)} \right], \\ \end{aligned}$$

6 $$v_{t} + uv_{x} + vv_{y} + wv_{z} = \nu v_{zz} + k_{0} \left( {v_{zzt} + vv_{zzy} + wv_{zzz} - v_{y} v_{zz} - v_{z} w_{zz} - 2v_{z} v_{yz} - 2w_{z} v_{zz} } \right),$$

7 $$T_{t} + uT_{x} + vT_{y} + wT_{z} = \alpha T_{zz} + \frac{{\left( {\rho c} \right)_{p} }}{{\left( {\rho c} \right)_{f} }}\left( {D_{B} \left( {T_{z} C_{z} } \right) + \frac{{D_{T} }}{{T_{\infty } }}\left( {T_{z} } \right)^{2} } \right) - \frac{1}{{\rho c_{p} }}\left( {q_{r} } \right)_{z} ,$$

8 $$C_{t} + uC_{x} + vC_{y} + wC_{z} = D_{B} \left( {C_{zz} } \right) + \frac{{D_{T} }}{{T_{\infty } }}\left( {T_{zz} } \right),$$

9 $$N_{t} + uN_{x} + vN_{y} + wN_{z} + \frac{{bW_{c} }}{{\left( {C_{w} - C_{\infty } } \right)}}\left[ {\partial_{z} \left( {NC_{z} } \right)} \right] = D_{m} \partial_{z} \left( {N_{z} } \right).$$

The radiative heat flux is expressed as

10 $$q_{r} = - \frac{{4\sigma^{*} }}{{3k^{*} }}D_{z} T^{4} = - \frac{{16\sigma^{*} }}{{3k^{*} }}T^{3} \left( {D_{z} T} \right).$$

Then expression (7) becomes

11 $$T_{t} + uT_{x} + vT_{y} + wT_{z} = \alpha_{m} T_{zz} + \frac{{\left( {\rho c} \right)_{p} }}{{\left( {\rho c} \right)_{f} }}\left( {D_{B} \left( {T_{z} C_{z} } \right) + \frac{{D_{T} }}{{T_{\infty } }}\left( {T_{zz} } \right)^{2} } \right) + \frac{1}{{\rho c_{p} }}\frac{{16\sigma^{*} }}{{3k^{*} }}\partial_{z} \left( {T^{3} T_{z} } \right).$$

The subjected boundary constraints are

12 $$\left. {\begin{array}{*{20}l} {u = U_{w} = \frac{ax}{{1 - ct}},\,v = V_{w} = \frac{by}{{1 - ct}},\,w = 0,\, - k\,T_{z} = h_{f} \left( {T_{w} - T} \right),\, - D_{B} C_{z} = h_{g} \left( {C_{w} - C} \right),} \hfill \\ { - D_{m} N_{z} = h_{m} \left( {N_{w} - N} \right)\,,at\,z = 0,\,T_{w} = T_{0} + a_{1}^{*} x,C_{w} = C_{0} + a_{2}^{*} x,N_{w} = N_{0} + a_{3}^{*} x,} \hfill \\ {u \to 0,\,v \to 0,\,T = T_{0} + d_{1}^{*} x,\,C = C_{0} + d_{2}^{*} x,\,N = N_{0} + d_{3}^{*} x,\,as\,z \to \infty ,} \hfill \\ \end{array} } \right\}.$$

In expressions (4)-(12), the components along *x, y,* and *z* axes are denoted as $$u\,,v$$ and $$w$$ respectively, $$\left( {\left( {\rho c} \right)_{p} } \right)$$ the heat capacity of nanoparticles, $$\left( {\rho_{f} } \right)$$ is the fluid density, $$\left( {\rho_{m} } \right)$$ density of microorganisms, $$\left( {\beta^{**} } \right)$$ volumetric expansion of the liquid, $$\left( \mu \right)$$ dynamic viscosity, $$\left( {\nu = \frac{\mu }{{\rho_{f} }}} \right)$$ kinematic viscosity,$$\left( {\left( {\rho c} \right)_{f} } \right)$$ heat capacity of base fluid, $$\left( \gamma \right)$$ the average volume of the microorganisms, $$\left( {q_{r} } \right)$$ radiative heat flux, $$\left( {\rho_{f} } \right)$$ density of the base fluid, $$\left( {\sigma^{*} } \right)$$ Stephan Boltzmann constant,$$\left( {k^{*} } \right)$$ absorption coefficient, $$\left( N \right)$$ microorganisms, $$\left( {N_{w} ,N_{\infty } } \right)$$ microorganisms surface and microorganisms away from surface respectively, $$\left( {a_{1}^{*} ,a_{2}^{*} ,a_{3}^{*} ,d_{1}^{*} ,d_{2}^{*} ,d_{3}^{*} } \right)$$ are dimensionless constants,$$\left( {g^{*} } \right)$$ is gravity, $$\left( {\alpha_{m} = \frac{k}{{\left( {\rho_{f} } \right)_{f} }}} \right)$$ is thermal diffusivity, $$\left( T \right)$$ temperature, $$\left( {k_{0} = \frac{{\alpha_{1} }}{{\rho_{f} }}} \right)$$ is viscoelastic fluid constant,$$\left( C \right)$$ nanoparticles concentration, $$\left( {T_{w} } \right)$$ wall temperature, $$\left( {T_{\infty } } \right)$$ temperature at surface $$\left( {D_{B} } \right),$$ $$\left( {D_{T} } \right)$$ and $$\left( {D_{m} } \right)$$ are Brownian motion, thermophoresis effects and microorganisms diffusions respectively $$\left( {C_{w} } \right)$$ is the concentration at the stretchable sheet and $$\left( {C_{\infty } } \right)$$ is the concentration of nanofluid away from the surface.

To obtain the dimensionless forms of Eqs. (4)-(12), the following similarities are tested^12,39^:

13 $$\left. {\begin{array}{*{20}l} {u = \frac{ax}{{\left( {1 - ct} \right)}}f^{\prime } \left( \zeta \right),\,v = \frac{ay}{{\left( {1 - ct} \right)}}g^{\prime } \left( \zeta \right),\,w = - \sqrt {\frac{a\nu }{{\left( {1 - ct} \right)}}} \left( {f\left( \zeta \right) + g\left( \zeta \right)} \right),\zeta = \sqrt {\frac{a}{{\nu \left( {1 - ct} \right)}}} {\text{z}},} \hfill \\ {\theta \left( \zeta \right)\left( {T_{w} - T_{\infty } } \right) + T_{\infty } = T,\,\phi \left( \zeta \right)\left( {C_{w} - C_{\infty } } \right) + C_{\infty } = C,\,\chi \left( \zeta \right)\left( {N_{w} - N_{\infty } } \right) + N_{\infty } = N,} \hfill \\ \end{array} } \right\}.$$

The results of main equations are

14 $$\begin{aligned} & f^{\prime \prime \prime } - f^{\prime 2} - S\left( {\frac{\zeta }{2}f^{\prime \prime } + f^{\prime}} \right) + \left( {f + g} \right)f^{\prime \prime } - \beta \left( \begin{gathered} - S\left( {\frac{\zeta }{2}f^{iv} + 2f^{\prime \prime \prime } } \right) + \left( {f + g} \right)f^{iv} \hfill \\ + \left( {f^{\prime \prime } + g^{\prime \prime } } \right)f^{\prime \prime } - 2\left( {f^{\prime } + g^{\prime } } \right)f^{\prime \prime \prime } \hfill \\ \end{gathered} \right) \\ & \quad + \;\lambda \left[ {\theta - Nr\phi - Nc\chi } \right] = 0, \\ \end{aligned}$$

15 $$g^{\prime \prime \prime } - g^{\prime 2} - S\left( {\frac{\zeta }{2}f^{\prime \prime } + f^{\prime } } \right) + \left( {f + g} \right)g^{\prime \prime } - \beta \left( \begin{gathered} - S\left( {\frac{\zeta }{2}g^{iv} + 2g^{\prime \prime \prime } } \right) + \left( {f + g} \right)g^{iv} \hfill \\ + \left( {g^{\prime \prime } - f^{\prime \prime } } \right)g^{\prime \prime } - 2\left( {f^{\prime } + g^{\prime } } \right)g^{\prime \prime \prime } \hfill \\ \end{gathered} \right)\, = 0,$$

16 $$\left( {1 + \frac{4}{3}Rd} \right)\theta^{\prime \prime } + Pr\left( {\left( {f + g} \right)\theta^{\prime } - f^{\prime } \theta - S_{1} f^{\prime } + Nb\theta^{\prime } \phi^{\prime } + Nt\theta^{\prime 2} - S\left( {\frac{\zeta }{2}\theta^{\prime } - \theta } \right)} \right) = 0,$$

17 $$\phi^{\prime \prime } + LePr\left( {\left( {f + g} \right)\phi^{\prime } - f^{\prime } \phi - S_{2} f^{\prime } - S\left( {\frac{\zeta }{2}\phi^{\prime } - \phi } \right)} \right) + \left( {\frac{Nt}{{Nb}}} \right)\theta^{\prime \prime } = 0,$$

18 $$\chi^{\prime \prime } + Lb\chi^{\prime } \left( {f + g} \right) - LbS_{3} f^{\prime } - Pe\left[ {\phi^{\prime \prime } \left( {\chi + \delta_{1} } \right) + \chi^{\prime } \phi^{\prime } } \right] = 0,$$

with

19 $$\left. {\begin{array}{*{20}l} {f\left( 0 \right) = 0,\,g\left( 0 \right) = 0,\,f^{\prime } \left( 0 \right) = 1,\,g^{\prime } \left( 0 \right) = \alpha ,\,\theta^{\prime } \left( 0 \right) = - \gamma_{1} \left( {1 - S_{1} - \theta \left( 0 \right)} \right),} \hfill \\ {\phi^{\prime } = - \gamma_{2} \left( {1 - S_{2} - \phi \left( 0 \right)} \right),\chi^{\prime } = - \gamma_{3} \left( {1 - S_{3} - \chi \left( 0 \right)} \right),\,at\,\zeta \to 0,} \hfill \\ {f^{\prime } \to 0,\,g^{\prime } \to 0,\,\theta \to 0,\,\phi \to 0,\,\chi \to 0,\;as\;\zeta \to \infty } \hfill \\ \end{array} } \right\},$$

where $\left( S \right)$ unsteadiness parameter, $\left( \beta \right)$ viscoelastic fluid parameter, $\left( {Nr} \right)$ buoyancy parameter, $\left( {Nc} \right)$ bio-convection Rayleigh parameter,$\left( \lambda \right)$ mixed convective number,$\left( {Nt} \right)$ thermophoresis parameter, $\left( \alpha \right)$ ratio parameter,$\left( {Nb} \right)$ Brownian motion number,$\left( {Le} \right)$ Lewis number,$\left( {Rd} \right)$ radiation parameter,$\left( {Pr} \right)$ Prandtl parameter,$\left( {\delta_{1} } \right)$ microorganisms concentration difference number,$\left( {Lb} \right)$ bioconvection Lewis number,$\left( {Pe} \right)$ Peclet number,$\left( {\gamma_{1} } \right)$ thermal Biot parameter, $\left( {\gamma_{2} } \right)$ solutal Biot parameter, $\left( {\gamma_{3} } \right)$ stratification Biot number,$\left( {S_{1} } \right)$ thermally stratified parameter,$\left( {S_{2} } \right)$ solutal stratified variable, and $\left( {S_{3} } \right)$ motile stratification number which are mathematically related in following forms:

20 $$\left. {\begin{array}{*{20}l} {S\left( { = \frac{c}{a},} \right)\,\beta \left( { = \frac{{K_{0} a}}{{\nu \left( {1 - ct} \right)}}} \right),Nr\left( { = \frac{{(\rho_{p} - \rho_{f} )(C_{w} - C_{\infty } )}}{{\left( {T_{w} - T_{\infty } } \right)\beta^{**} \rho_{f} (1 - C_{\infty } )}}} \right),\alpha \left( { = \frac{b}{a}} \right),Le\left( { = \frac{{\alpha_{m} }}{{D_{B} }}} \right),} \hfill \\ {Nc\left( { = \frac{{\gamma (\rho_{m} - \rho_{f} )(N_{w} - N_{\infty } )}}{{\rho_{f} (1 - C_{\infty } )\left( {T_{w} - T_{\infty } } \right)\,\beta^{**} }}} \right),\,\lambda \left( { = \frac{{\beta^{**} g^{*} \left( {T_{w} - T_{\infty } } \right)\left( {1 - C_{\infty } } \right)\left( {1 - ct} \right)^{2} }}{{a^{2} x}}} \right),\,} \hfill \\ {Nt\left( { = \frac{{\left( {\rho c} \right)_{p} D_{T} \left( {T_{w} - T_{\infty } } \right)}}{{\left( {\rho c} \right)_{f} T_{\infty } \nu }}} \right),\,Nb\left( { = \frac{{\left( {\rho c} \right)_{p} D_{B} \left( {C_{w} - C_{\infty } } \right)}}{{\left( {\rho c} \right)_{f} \nu }}} \right),Rd\left( { = \frac{{4\sigma^{*} T_{\infty }^{3} }}{{kk^{*} }}} \right),} \hfill \\ {\Pr \left( { = \frac{\nu }{{\alpha_{m} }}} \right),\delta_{1} \left( { = \frac{{N_{\infty } }}{{N_{w} - N_{\infty } }}} \right),{\text{Lb}}\left( { = \frac{\nu }{{D_{m} }}} \right),Pe\left( { = \frac{{bW_{c} }}{{D_{m} }}} \right)\,,\gamma_{1} \left( { = \left( {\frac{{h_{f} }}{k}} \right)\sqrt {\frac{{\nu \left( {1 - ct} \right)}}{a}} } \right),} \hfill \\ {\gamma_{2} \left( { = \left( {\frac{{h_{g} }}{k}} \right)\sqrt {\frac{{\nu \left( {1 - ct} \right)}}{a}} } \right),\gamma_{3} \left( { = \left( {\frac{{h_{m} }}{k}} \right)\sqrt {\frac{{\nu \left( {1 - ct} \right)}}{a}} } \right),S_{1} \left( { = \frac{{d_{1}^{*} }}{{a_{1}^{*} }}} \right),S_{2} \left( { = \frac{{d_{2}^{*} }}{{a_{2}^{*} }}} \right),S_{3} \left( { = \frac{{d_{3}^{*} }}{{a_{3}^{*} }}} \right)} \hfill \\ \end{array} } \right\}.$$

The physical quantities such as Nusselt number is introduced as^5,12–57^.

21 $$Nu_{x} = \left. { - \frac{x}{{\left( {T - T_{\infty } } \right)}}\frac{\partial T}{{\partial z}}} \right|_{z = 0} .$$

Sherwood number and microorganism’s density number are addressed as

22 $$\begin{aligned} & {\text{Sn}}_{x} = \frac{{xj_{m} }}{{D_{T} \left( {C_{w} - C_{\infty } } \right)}}, \\ & {\text{Nn}}_{x} = \frac{{xj_{n} }}{{D_{m} \left( {N_{w} - N_{\infty } } \right)}}. \\ \end{aligned}$$

From above relations (21) and (22), we obtain the following dimensionless forms:

23 $$\begin{aligned} & {\text{Re}}^{{\frac{ - 1}{2}}} Nu = - \theta^{\prime } \left( 0 \right), \\ & {\text{Re}}^{{\frac{ - 1}{2}}} Sn = - \theta^{\prime } \left( 0 \right), \\ & Nn{\text{Re}}_{x}^{{ - \frac{1}{2}}} = - \chi^{\prime } \left( 0 \right). \\ \end{aligned}$$

The local Reynolds number is ${\text{Re}} = \frac{ux}{v}$.

### Numerical approach

Because of its accuracy and performance, the bvp4c scheme numerically computes the expressions (14)-(18) with boundary conditions (19). Initially, coupled non-linear ODEs are converted to first-order initial-value problems using the following procedure:

24 $$\left. {\begin{array}{*{20}l} {f = z_{1} ,f^{\prime } = z_{2} ,f^{\prime \prime } = z_{3} ,f^{\prime \prime \prime } = z_{4} ,f^{iv} = z_{4}^{\prime } ,{\text{g}} = z_{5} ,{\text{g}}^{\prime } = z_{6} ,} \hfill \\ {{\text{g}}^{\prime \prime } = z_{7} ,{\text{g}}^{\prime \prime \prime } = z_{8} ,{\text{g}}^{iv} = z_{8}^{\prime } ,\theta = z_{9} ,\theta^{\prime } = z_{10} ,\theta^{\prime \prime } = z_{10}^{\prime } ,\phi = z_{11} ,} \hfill \\ {\phi^{\prime } = z_{12} ,\phi^{\prime \prime } = z^{\prime }_{12} ,\chi = z_{13} ,\chi^{\prime } = z_{14} ,\chi^{\prime \prime } = z^{\prime } z^{\prime}_{14} } \hfill \\ \end{array} } \right\}$$

25 $$\left. {\begin{array}{*{20}l} {z_{4}^{^{\prime}} = \frac{\begin{gathered} - z_{4} + z_{2}^{2} + S\left( {0.5\zeta z_{3} + z_{2} } \right) - \left( {z_{1} + z_{5} } \right)z_{3} + \beta \left( \begin{gathered} - 2Sz_{4} + \left( {z_{3} + z_{7} } \right)z_{3} \hfill \\ - 2\left( {z_{2} + z_{6} } \right)z_{4} \hfill \\ \end{gathered} \right) \hfill \\ - \lambda \left[ {z_{9} - Nrz_{11} - Ncz_{13} } \right] \hfill \\ \end{gathered} }{{\beta \left( {S\left( {0.5\zeta } \right) - \left( {z_{1} + z_{5} } \right)} \right)}},} \hfill \\ {z_{8}^{^{\prime}} = \frac{\begin{gathered} - z_{8} + z_{6}^{2} + S\left( {0.5\zeta z_{3} + z_{2} } \right) - \left( {z_{1} + z_{5} } \right)z_{7} + \left( {z_{7} - z_{3} } \right)z_{7} - 2\left( {z_{2} + z_{6} } \right)z_{8} \hfill \\ + \beta \left( \begin{gathered} - 2sz_{8} + \left( {z_{7} + z_{3} } \right)z_{7} \hfill \\ - 2\left( {z_{2} - z_{6} } \right)z_{8} \hfill \\ \end{gathered} \right) \hfill \\ \end{gathered} }{{\beta \left( {S\left( {0.5\zeta } \right) - \left( {z_{1} + z_{5} } \right)} \right)}}\,,} \hfill \\ {z_{10}^{^{\prime}} = - Pr\left( {\left( {z_{1} + z_{5} } \right)z_{10} - z_{2} z_{9} - S_{1} z_{2} + Nbz_{10} z_{12} + Ntz_{10}^{2} - S\left( {0.5\zeta z_{10} - z_{9} } \right)} \right),} \hfill \\ {z_{12}^{^{\prime}} = - Le\Pr \left( {\left( {z_{1} + z_{5} } \right)z_{12} - z_{2} z_{11} - S_{2} z_{2} - S\left( {0.5\zeta z_{12} - z_{11} } \right)} \right) + \left( {\frac{Nt}{{Nb}}} \right)z^{\prime}_{10} ,} \hfill \\ {z_{14}^{^{\prime}} = Pe\left[ {z_{12}^{^{\prime}} \left( {z_{13} + \delta_{1} } \right) + z_{12} z_{14} } \right] - Lbz_{1} z_{14} + LbS_{3} z_{2} ,} \hfill \\ {z_{1} \left( 0 \right) = 0,\,z_{5} \left( 0 \right) = 0,\,z_{2} \left( 0 \right) = 1,\,z_{6} \left( 0 \right) = \alpha ,\,z_{10} \left( 0 \right) = - \gamma_{1} \left( {1 - S_{1} - z_{9} \left( 0 \right)} \right),} \hfill \\ {z_{12} = - \gamma_{2} \left( {1 - S_{2} - z_{11} \left( 0 \right)} \right),z_{14} = - \gamma_{3} \left( {1 - S_{3} - z_{13} \left( 0 \right)} \right),\,at\,\zeta \to 0,} \hfill \\ {z_{2} \to 0,\,z_{6} \to 0,\,\,z_{9} \to 0,\,\,z_{11} \to 0,\,\,z_{13} \to 0, as\,\,\zeta \to \infty } \hfill \\ \end{array} } \right\}.$$

## Results and discussion

In the present section, our prime motive is to scrutinize the physical influence of the numerical model through graphical illustrations. The rheological behavior of significant parameters like radiation parameter $Rd$, viscoelastic fluid parameter $\beta$, unsteady parameter $S$, mixed convection parameter $\lambda$, Lewis number $Le$, bioconvection Lewis number $Lb$, ratio parameter $\alpha$, Peclet number $Pe$, Brownian motion parameter $Nb$, Prandtl number $\Pr$, thermal boot number $\gamma_{1}$, buoyancy ratio parameter $Nr$, thermophoresis parameter $Nt$, solutal Biot number $\gamma_{2}$, bioconvection Rayleigh number $Nc$ and stratification Biot number $\gamma_{3}$ for velocities, temperature, nanoparticles concentration, and motile microorganisms profiles is observed. The ranges of the different parameters like as,$0.1 \le Nc \le 1.2$,$0.0 \le \alpha \le 1.2$, $0.2 \le \gamma_{1} \le 0.8$, $0.0 \le S \le 0.6$, $1.0 \le Le \le 2.0$,$0.5 \le Nt \le 2.0$, $0.5 \le \Pr \le 2.0$, $0.1 \le Nr \le 1.5$, $0.5 \le Nb \le 2.0$, $0.1 \le \lambda \le 1.2$, $0.1 \le Rd \le 1.5$, $0.2 \le \gamma_{2} \le 0.8$, $0.2 \le \gamma_{3} \le 0.8$, $1.0 \le Lb \le 2.0$,$0.1 \le \beta \le 1.5$ and $0.1 \le {\text{Pe}} \le 1.0$ are analyzed.

**Figure 2:**
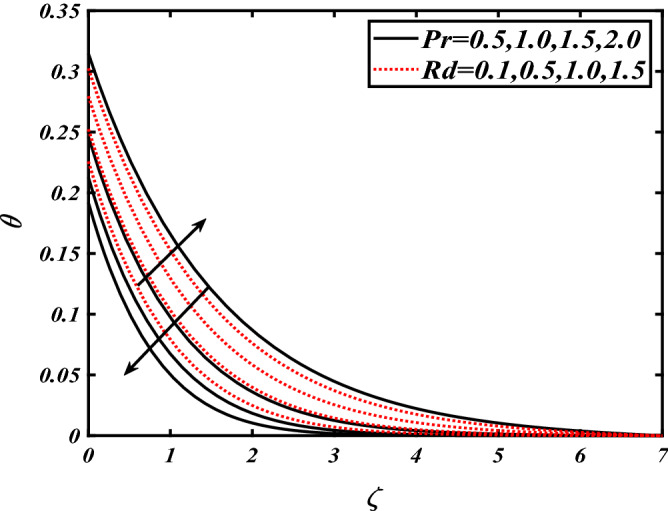
Illustration of $Pr\,and\,Rd$ over-temperature $\theta$ when $\alpha = 0.5,Nb = 0.2,Nt = 0.3,S = 0.5,\lambda = 0.5,\beta = 0.2$.

**Figure 3:**
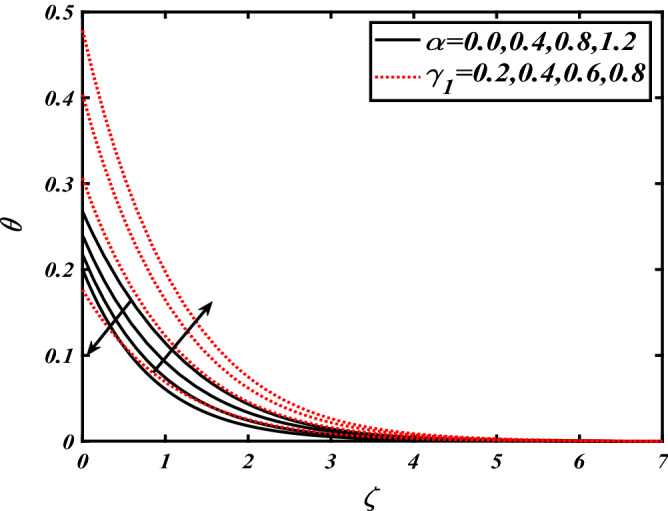
Illustration of $\alpha \,and\,\gamma_{1}$ over-temperature $\theta$ when $Pr = 2.0,{\text{Rd}} = 0.4,\gamma_{2} = 0.3,Nb = 0.2,Nt = 0.3,S = 0.5,\lambda = 0.5,\beta = 0.2$.

**Figure 4:**
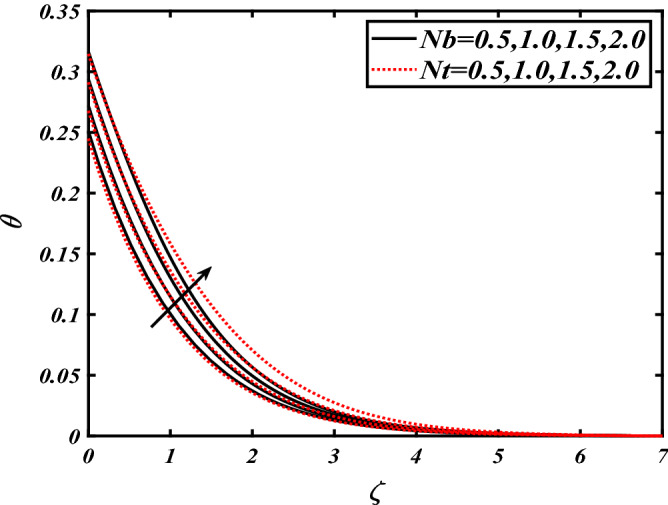
Illustration of $Nb\,and\,Nt$ over-temperature $\theta$ when $Pr = 2.0,{\text{Rd}} = 0.4,\gamma_{2} = 0.3,Nr = 0.2,Nc = 0.2,S = 0.5,\lambda = 0.5,\beta = 0.2$.

**Figure 5:**
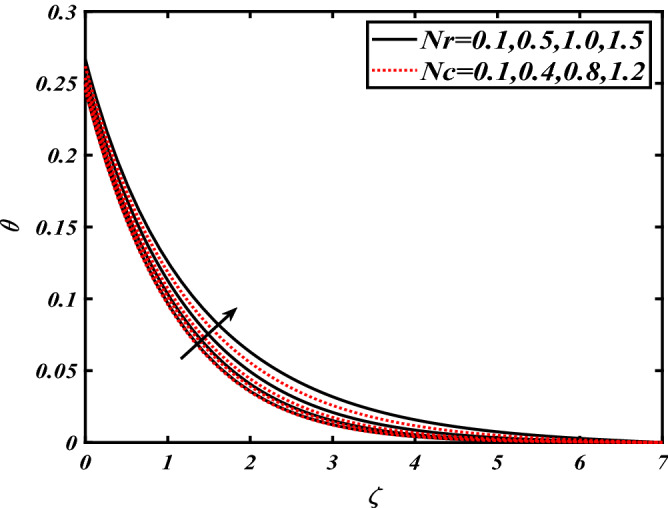
Illustration of $Nr\,and\,Nc$ overtemperature $\theta$ when $Pr = 2.0,{\text{Rd}} = 0.4,\gamma_{2} = 0.3,Nb = 0.2,Nt = 0.3,S = 0.5,\lambda = 0.5,\beta = 0.2$.

**Figure 6:**
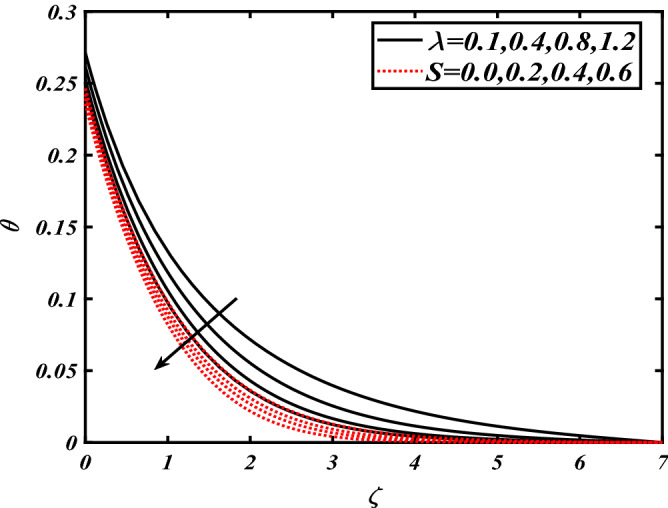
Illustration of $\lambda \,and\,S$ over-temperature $\theta$ when $Pr = 2.0,{\text{Rd}} = 0.4,\gamma_{2} = 0.3,Nb = 0.2,Nt = 0.3,\beta = 0.2$.

**Figure 7:**
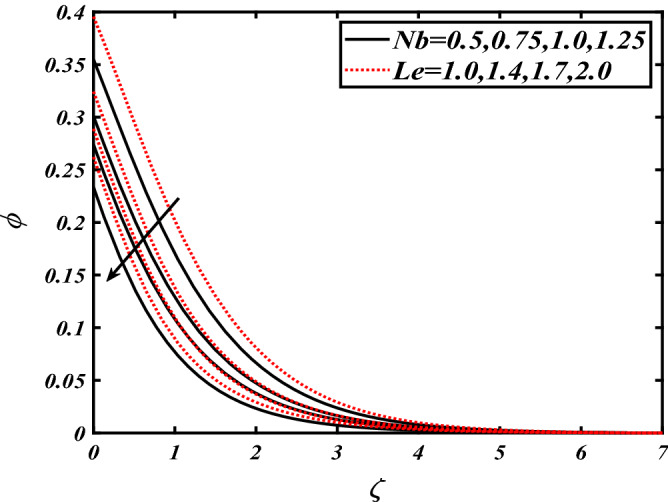
Illustration of $Nb\,and\,Le$ over nanoparticles concentration $\phi$ when $Pr = 2.0,{\text{Rd}} = 0.4,\gamma_{2} = 0.3,\gamma_{1} = 0.3,Nt = 0.3,S = 0.5,\lambda = 0.5,\beta = 0.2$.

**Figure 8:**
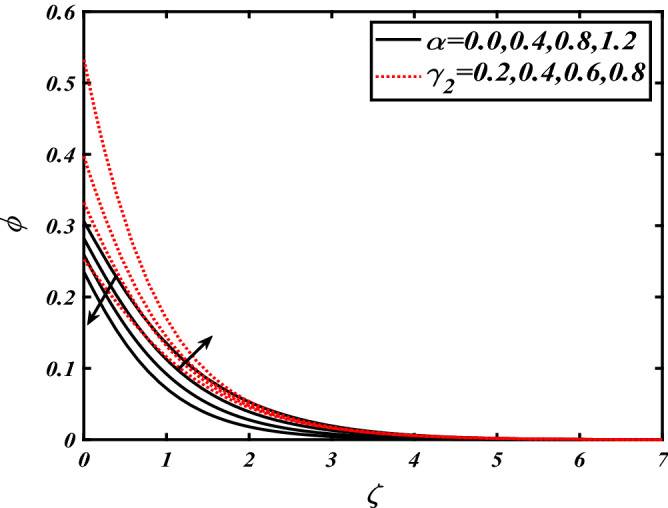
Illustration of $\alpha \,and\,\gamma_{2}$ over nanoparticles concentration $\phi$ when $Pr = 2.0,{\text{Rd}} = 0.4,\gamma_{1} = 0.3,Nt = 0.3,S = 0.5,\lambda = 0.5,\beta = 0.2$.

**Figure 9:**
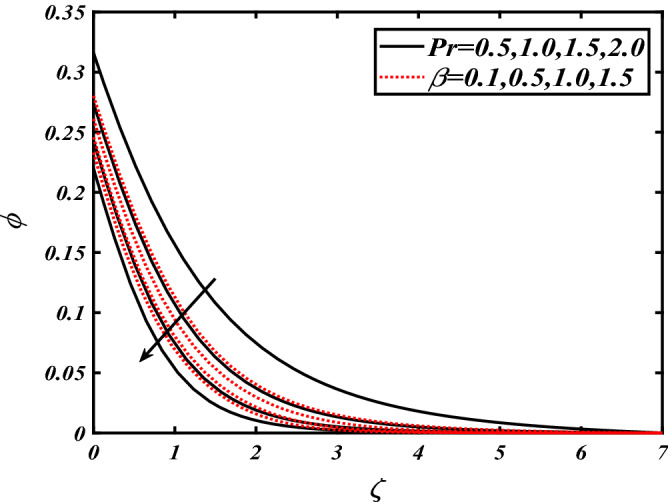
Illustration of $\Pr \,and\,\beta$ over nanoparticles concentration $\phi$ when ${\text{Rd}} = 0.4,\gamma_{2} = 0.3,\gamma_{1} = 0.3,Nt = 0.3,{\text{Nb}} = 0.2,{\text{Nr}} = 0.2,S = 0.5,\lambda = 0.5$.

**Figure 10:**
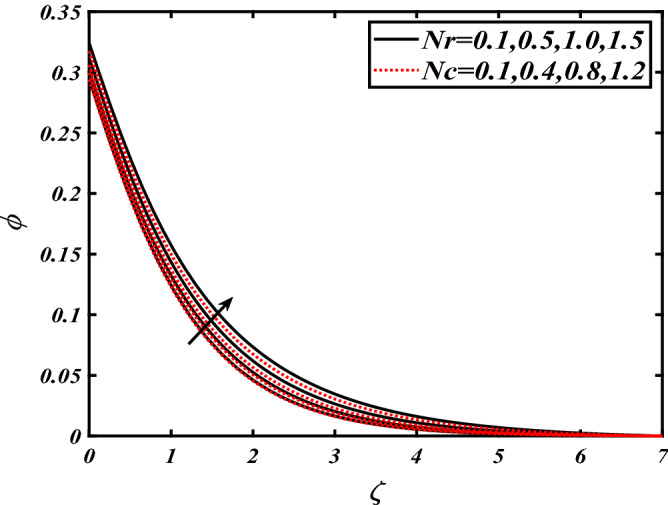
Illustration of $Nr\,and\,Nc$ over nanoparticles concentration $\phi$ when ${\text{Rd}} = 0.4,\gamma_{2} = 0.3,\gamma_{1} = 0.3,Nt = 0.3,{\text{Nb}} = 0.2,S = 0.5,\lambda = 0.5$.

**Figure 11:**
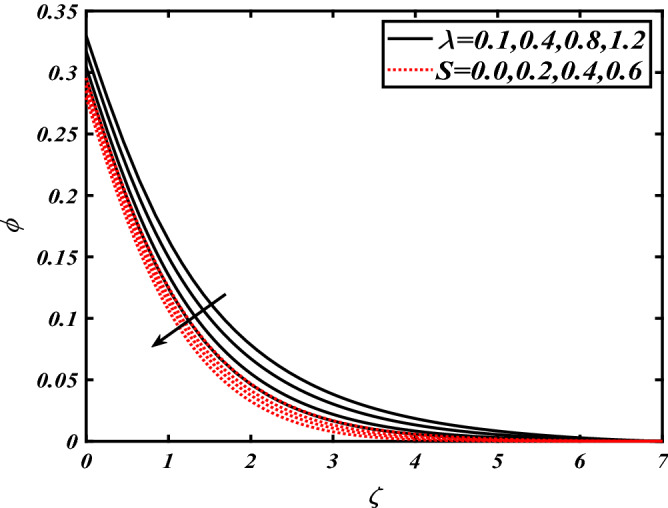
Illustration of $\lambda \,and\,S$ over nanoparticles concentration $\phi$ when ${\text{Rd}} = 0.4,\gamma_{2} = 0.3,\gamma_{1} = 0.3,Nt = 0.3,{\text{Nb}} = 0.2,{\text{Nr}} = 0.2$.

**Figure 12:**
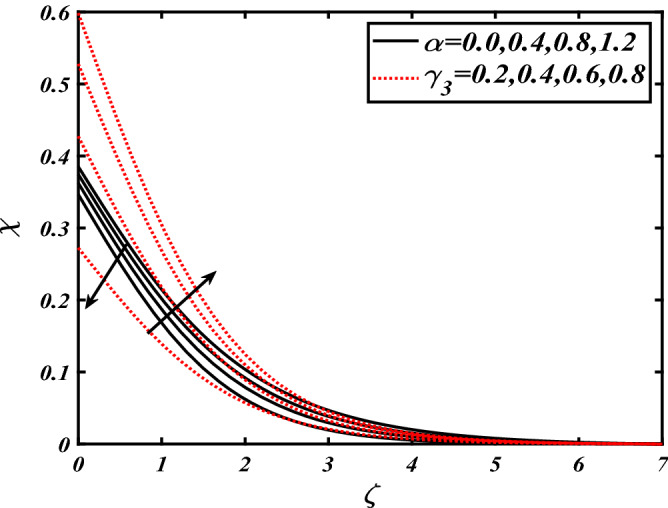
Illustration of $\alpha \,and\,\gamma_{3}$ over motile microorganisms $\chi$ when ${\text{Rd}} = 0.4,\gamma_{2} = 0.3,\gamma_{1} = 0.3,Nt = 0.3,{\text{Nb}} = 0.2,{\text{Nr}} = 0.2,S = 0.5,\lambda = 0.5,{\text{Pe}} = 0.1,{\text{Lb}} = 2.0$.

**Figure 13:**
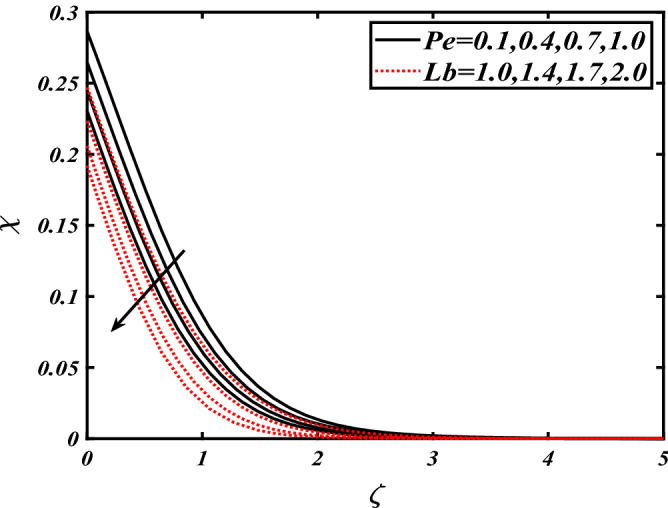
Illustration of $Pe\,and\,Lb$ over motile microorganisms $\chi$ when ${\text{Rd}} = 0.4,\gamma_{2} = 0.3,\gamma_{1} = 0.3,Nt = 0.3,{\text{Nb}} = 0.2,{\text{Nr}} = 0.2,S = 0.5,\lambda = 0.5$.

**Figure 14:**
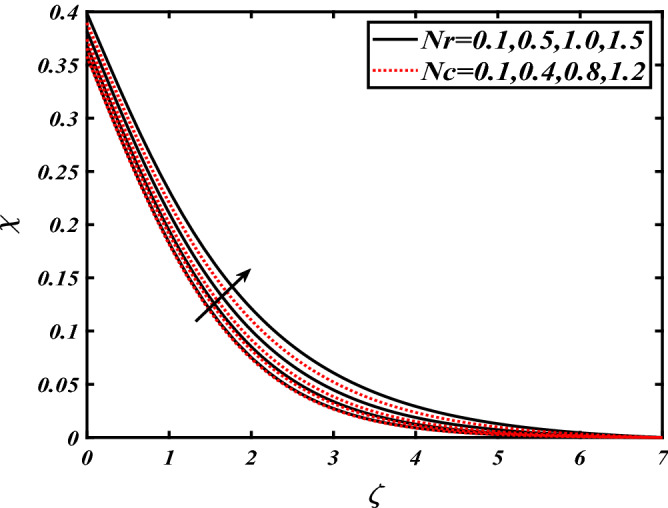
Illustration of $Nr\,and\,Nc$ over motile microorganisms $\chi$ when ${\text{Rd}} = 0.4,\gamma_{2} = 0.3,\gamma_{1} = 0.3,{\text{Nr}} = 0.2,S = 0.5,\lambda = 0.5,{\text{Pe}} = 0.1,{\text{Lb}} = 2.0$.

**Figure 15:**
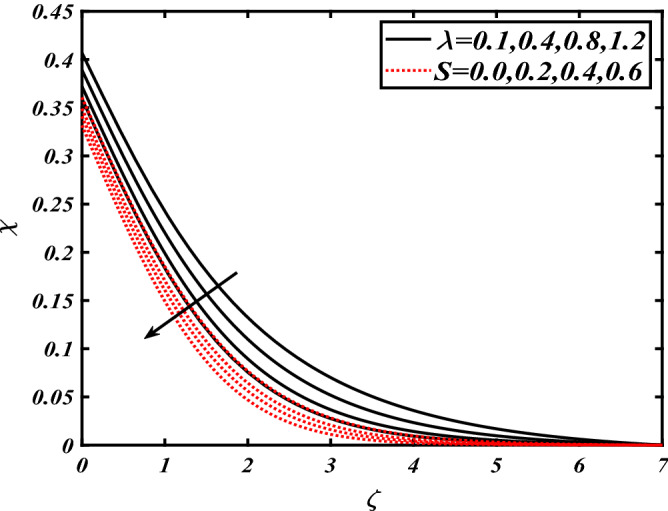
Illustration of $\lambda \,and\,S$ over motile microorganisms $\chi$ when ${\text{Rd}} = 0.4,\gamma_{2} = 0.3,\gamma_{1} = 0.3,{\text{Nr}} = 0.2,{\text{Pe}} = 0.1,{\text{Lb}} = 2.0$.

Thus Figs. 2, 3, 4, 5, 6, 7, 8, 9, 10, 11, 12, 13, 14 and 15 are drafted. The plot of temperature distribution $\theta \left( \zeta \right)$ for various variations Pr and $Rd$ is portrayed in Fig. 2. It is analyzed that the rise of thermal radiation parameter $Rd$ leads to an increment in temperature field $\theta \left( \zeta \right)$ and it is declining for Prandtl number Pr. Physically Pr is an inverse proportion to temperature. A larger Prandtl number Pr declines the thermal diffusivity which shows a decrement in the temperature of the fluid. Physically more heat is produced when thermal radiation is improved. The deteriorating behavior of the ratio parameter $\alpha$ and Biot number $\gamma_{1}$ against $\theta \left( \zeta \right)$ is delineated in Fig. 3. As anticipated, temperature distribution $\theta \left( \zeta \right)$ dwindles for progressive values of the ratio parameter $\alpha$. Furthermore, the temperature distribution $\theta \left( \zeta \right)$ is boomed up by rising values of thermal Biot number $\gamma_{1}$.Physically by increasing the values of thermal Biot number reduces the resistance for energy transport at the surface and as a result, more temperature is attained. Figure 4 intimates the illustration of $Nb$ and thermophoresis parameter $Nt$ via $\theta \left( \zeta \right)$. The temperature field $\theta \left( \zeta \right)$ is progressive for higher values of both parameters (Brownian motion parameter $Nb$&thermophoresis parameter $Nt$). Brownian movement is the random movement of fluid suspended molecules caused by a collision with the rapidly moving particles of the fluid. Physically in thermophoresis mechanisms i.e., the particles of fluid transport from a hot region to a cool region. The behavior of buoyancy ratio parameter $Nr$ and bioconvection Rayleigh number $Nc$ over-temperature filed $\theta \left( \zeta \right)$ is demonstrated in Fig. 5. In the current situation for higher variations of $Nr$ and bioconvection Rayleigh number $Nc$, temperature $\theta \left( \zeta \right)$ increases. Figure 6 discloses the roles of unsteady parameter $S$ and mixed convection parameter $\lambda$ versus thermal distribution $\theta \left( \zeta \right)$. The temperature distribution $\theta \left( \zeta \right)$ is truncated subjected to the higher deviation of $\lambda$ and unsteady number $S$. Figure 7 communicates the physical significance of the $Nb$ and $Le$ against concentration $\phi \left( \zeta \right)$. Contrasting behavior is observed in volumetric concentration $\phi \left( \zeta \right)$ against Lewis number $Le$ and Brownian motion parameter $Nb$. Physically enhancing values of Lewis number reduces the mass diffusivity. Thus energy profile diminishes. Figure 8 elucidates the influence of ratio parameter $\alpha$ and solutal Biot number $\gamma_{2}$ against volumetric concentration $\phi \left( \zeta \right)$. It is intimated that due to increment in solutal Biot number $\gamma_{2}$ the volumetric concentration $\phi \left( \zeta \right)$ is enhanced. Furthermore, we observed that the concentration curves also decreased for enhancement in the ratio parameter $\alpha$. The consequences of Pr and viscoelastic fluid parameter $\beta$ on volumetric concentration $\phi \left( \zeta \right)$ are communicated in Fig. 9. It is noted that the $\phi \left( \zeta \right)$ reduces by uplifting the variations of Prandtl number Pr and viscoelastic parameter $\beta$. Figure 10 is captured the vital role of bioconvection Rayleigh number $Nc$ and buoyancy ratio parameter $Nr$ via concentration field $\phi \left( \zeta \right)$. The concentration field $\phi \left( \zeta \right)$ is knocked down due to progressive values of buoyancy ratio parameter $Nr$ and bioconvection Rayleigh number $Nc$. Figure 11 determines the inspiration of $\lambda$ an unsteady number $S$ on volumetric concentration $\phi \left( \zeta \right)$. A retarded concentration has been examined with the variation of mixed convection parameter $\lambda$ and unsteady number $S$. It can be seen that the concentration declines with an enhancing mixed convection parameter $\lambda$. The concentration field also reduces for different variations of the unsteady parameter $S$. Figure 12 constitutes the outcomes of mixed convection parameter $\lambda$ and stratification Biot number $\gamma_{3}$ on $\chi \left( \zeta \right)$. The motile microorganism $\chi \left( \zeta \right)$ upsurges by varying values of stratification Biot number $\gamma_{3}$. The curve of motile microorganisms $\chi \left( \zeta \right)$ maps down for growing mixed convection parameter $\lambda$. Figure 13 is mapped to delineate the significance of Peclet number ${\text{Pe}}$ and bioconvection Lewis number $Lb$ over motile microorganism’s concentration $\chi \left( \zeta \right)$. Motile microorganism’s field $\chi \left( \zeta \right)$ diminishes when the Peclet number ${\text{Pe}}$ enhances. Furthermore, a decreasing trend is noted by variations of bioconvection Lewis number $Lb$.Physically, when we enhance the Peclet number Pe, the diffusion of microorganisms reduces which causes a reduction in the microorganism’s profile. Figure 14 reports the diversity of motile microorganisms field $\chi \left( \zeta \right)$ for several estimations of buoyancy ratio parameter $Nr$ and bioconvection Rayleigh number $Nc$. Improvement in both parameters divulges the enhancing conduct for motile microorganisms $\chi \left( \zeta \right)$. Figure 15 depicts a variety of mixed convective parameter $\lambda$ and unsteady number $S$ against motile microorganisms. Interestingly motile microorganisms $\chi \left( \zeta \right)$ are declining function for mixed convection parameter $\lambda$ and unsteady parameter $S$.To illustrate the inspiration of prominent flow parameters versus local Nusselt and Sherwood numbers, Table 1 is captured. The features of the involved parameters are deeply analyzed. Local Sherwood and Nusselt numbers are enhanced by growing variation of Prandtl number Pr. The performance of prominent parameters against the density of motile microorganisms is divulged in Table 2. It is analyzed that the density number of motile microorganisms shows the declining trend for higher variations of bioconvection Lewis number $Lb$ and Peclet number Pe.

**Table 1:** Tabulations of $- \theta^{\prime } \left( 0 \right)$ and $- \phi^{\prime } \left( 0 \right)$ with variations of $\alpha$, $S$, $\beta$, Pr, $Nb$, $Nt$, $Le$, $\lambda$, $Nr$, $Nc$ and $\gamma_{1}$.

$S$	$\beta$	Pr	$Nb$	$Nt$	$Le$	$\lambda$	$Nr$	$Nc$	$\gamma_{1}$	$- \theta^{\prime } \left( 0 \right)$	$- \phi^{\prime } \left( 0 \right)$
1.0	0.0	1.2	0.2	0.3	2.0	0.1	0.2	0.2	0.3		
0.1 0.4 0.8	0.0	1.2	0.2	0.3	2.0	0.1	0.2	0.2	0.3	0.2406 0.2434 0.2480	0.2532 0.2520 0.2518
1.0	0.0 0.4 0.8	1.2	0.2	0.3	2.0	0.1	0.2	0.2	0.3	0.2497 0.2503 0.2508	0.2515 0.2523 0.2529
1.0	0.0	1.0 2.0 3.0	0.2	0.3	2.0	0.1	0.2	0.2	0.3	0.2461 0.2595 0.2660	0.2486 0.2609 0.2673
1.0	0.0	1.2	0.1 0.4 0.8	0.3	2.0	0.1	0.2	0.2	0.3	0.2503 0.2495 0.2485	0.2343 0.2607 0.2650
1.0	0.0	1.2	0.2	0.1 0.5 1.0	2.0	0.1	0.2	0.2	0.3	0.2506 0.2494 0.2479	0.2634 0.2407 0.2138
1.0	0.0	1.2	0.2	0.3	1.0 1.4 1.8	0.1	0.2	0.2	0.3	0.2247 0.2297 0.2307	0.2344 0.2432 0.2494
1.0	0.0	1.2	0.2	0.3	2.0	0.2 0.4 0.8	0.2	0.2	0.3	0.2405 0.2513 0.2623	0.2513 0.2612 0.2714
1.0	0.0	1.2	0.2	0.3	2.0	0.1	0.3 0.4 0.5	0.2	0.3	0.2357 0.2349 0.2334	0.2515 0.2494 0.2448
1.0	0.0	1.2	0.2	0.3	2.0	0.1	0.2	0.3 0.4 0.5	0.3	0.2256 0.2149 0.2066	0.2442 0.2424 0.2401
1.0	0.0	1.2	0.2	0.3	2.0	0.1	0.2	0.2	0.1 0.5 1.0	0.0939 0.3740 0.5927	0.2628 0.2442 0.2303

**Table 2:** Tabulations of $- \chi^{\prime } (0)$ with variations of $\alpha$, $S$, $\beta$, $\lambda$, $Nr$, $Nc$, $Pe$, $Lb$ and $\gamma_{3}$.

$S$	$\beta$	$\lambda$	$Nr$	$Nc$	$Pe$	$Lb$	$\gamma_{3}$	$- \chi^{\prime } (0)$
1.0	0.0	0.1	0.2	0.2	0.1	2.0	0.3	
0.1 0.4 0.8	0.0	0.1	0.2	0.2	0.1	2.0	0.3	0.2574 0.2567 0.2559
1.0	0.0 0.4 0.8	0.1	0.2	0.2	0.1	2.0	0.3	0.2554 0.2561 0.2565
1.0	0.0	0.2 0.4 0.8	0.2	0.2	0.1	2.0	0.3	0.2658 0.2743 0.2840
1.0	0.0	0.1	0.3 0.4 0.5	0.2	0.1	2.0	0.3	0.2635 0.2657 0.2701
1.0	0.0	0.1	0.2	0.3 0.4 0.5	0.1	2.0	0.3	0.2725 0.2653 0.2513
1.0	0.0	0.1	0.2	0.2	0.2 0.5 0.8	2.0	0.3	0.2547 0.2601 0.2628
1.0	0.0	0.1	0.2	0.2	0.1	1.0 1.4 1.8	0.3	0.2371 0.2466 0.2528
1.0	0.0	0.1	0.2	0.2	0.1	2.0	0.1 0.5 1.0	0.0948 0.3907 0.6408

## Conclusions

The physical representation of the three-dimensional time-dependent flow of viscoelastic nanofluid via a stretched surface in the presence of microorganisms has been examined. The viscoelastic fluid contains both elastic and viscous properties. Physical results of the various parameters are described by using different graphs. Volumetric fraction of nanoparticles and the temperature distribution are significantly retarded as mounting values of unsteadiness parameter while temperature and concentration profiles are elevated for the rising values of thermophoresis parameter. The concentration profile shows favorable reduction due to variation in Brownian motion parameter while the inverse pattern is observed for bioconvection Rayleigh number. The decreasing trend over-concentration of nanoparticles is to be noticed as intensifying values of the viscoelastic parameter. The volumetric concentration of nanoparticles reduces by boosting the Prandtl number. Increased bioconvection Lewis and Peclet populations decrease the motile microorganism field. In biomedicine and automobile solutions, electrochemistry, and mechanics, viscoelastic fluid play a vital role^58–60^. The significant applications in the fields of nanotechnology, bacteriology, bioengineering, petroleum, metalworking, biofuels, and engineering problems, for the research of nanofluid, has piqued the interest of researchers and scientist in recent years. The latest results are precise and pure, making them more useful in engineering fields.

## List of symbols

*u*, *v*, *w*: Components of velocity (m s^−1^)
$\delta_{1}$: Microorganisms difference number
$$\alpha_{m}$$: Thermal diffusivity (m^2^ s^−1^)
*x*, *y*, *z*: Space coordinates (m)
$\left( {\rho c} \right)_{p}$: Heat capacity of nanoparticles (J kg^−3^ K^−1^)
$\gamma_{1}$: Thermal Biot number
$Lb$: Bio convection Lewis number
$$\alpha$$: Ratio parameter
$\beta$: Viscoelastic fluid parameter
$S_{1}$: Thermally stratified parameter
$$\nu$$: Kinematic viscosity (m^2^ s^−1^)
$Nn$: Microorganism density number
$\left( {\rho c} \right)_{f}$: Heat capacity of base fluid (J kg^−3^ K^−1^)
$S_{2}$: Solutal stratified parameter
$S_{3}$: Microorganisms stratified parameter
$$D_{m}$$: Microorganisms diffusivity (m^2^ s^−1^)
$$Pr$$: Prandtl parameter
$\lambda$: Mixed convective number
b: Chemotaxis constant (m)
$\rho_{f}$: Fluid’s density (kg m^−3^)
$Nr$: Buoyancy-ratio parameter
$\gamma_{2}$: Solutal Biot number
$Nb$: Brownian motion number
$Nc$: Bio convection Rayleigh number
$W_{c}$: Speed of cell swimming (m s^−1^)
$$Nt$$: Thermophoresis number
$$Le$$: Lewis number
$\gamma_{3}$: Microorganisms stratification Biot number
$$Pe$$: Peclet parameter
*Nu*: Nusselt number
*Sn*: Sherwood number
$$D_{B}$$: Brownian diffusivity (m^2^ s^−1^)
$$D_{T}$$: Thermophoresis diffusivity (m^2^ s^−1^)
*N*: Microorganisms
$$\begin{gathered} a_{1}^{*} ,a_{2}^{*} ,a_{3}^{*} , \hfill \\ d_{1}^{*} ,d_{2}^{*} ,d_{3}^{*} \hfill \\ \end{gathered}$$: Dimensionless constants
